# Selective T_3_–T_4_ sympathicotomy versus gray ramicotomy on outcome and quality of life in hyperhidrosis patients: a randomized clinical trial

**DOI:** 10.1038/s41598-021-96972-7

**Published:** 2021-09-02

**Authors:** Vicente Vanaclocha, Ricardo Guijarro-Jorge, Nieves Saiz-Sapena, Manuel Granell-Gil, José María Ortiz-Criado, Juan Manuel Mascarós, Leyre Vanaclocha

**Affiliations:** 1 Department of Neurosurgery, Hospital General Universitario de Valencia and Department of Surgery, Faculty of Medicine, University of Valencia, Avenida Blasco Ibáñez 15, 46010 Valencia, Spain; 2 Department of Thoracic Surgery, Hospital General Universitario de Valencia and Department of Surgery, Faculty of Medicine, University of Valencia, Valencia, Spain; 3 Department of Anesthesiology, Hospital General Universitario de Valencia, Valencia, Spain; 4 Instituto de Medicina Legal de Valencia (IMLV) and Department of Anatomy, Faculty of Medicine, Catholic University St. Vincent Martyr of Valencia, Valencia, Spain; 5 Mathematician With a Master in Statistics, Department of Statistics, Research Foundation, Hospital General Universitario, Valencia, Spain; 6 Medical School, University College London, London, UK

**Keywords:** Neuroscience, Diseases, Health care, Medical research

## Abstract

Compensatory hyperhidrosis is the leading cause of patients' dissatisfaction after thoracic sympathicotomy. The study aimed to reduce compensatory hyperhidrosis to increase patients’ satisfaction. A prospective randomized study on palmar hyperhidrosis, May 2016–September 2019. Twenty-one patients T_3_–T_4_ sympathicotomy and 21 T_3_–T_4_ gray ramicotomy. Data prospectively collected. Analysis at study's end. Focus on the sweating, temperature, quality of life baseline and postoperatively, compensatory hyperhidrosis, hand dryness, patients' satisfaction, and if they would undergo the procedure again and recommend it. No baseline differences between groups. Hyperhidrosis was controlled postoperatively in all patients. No mortality, serious complications, or recurrences. Sympathicotomy worse postoperative quality of life (49.05 (SD: 15.66, IR: 35.50–63.00) versus ramicotomy 24.30 (SD: 6.02, IR: 19.75–27.25). After ramicotomy, some residual sweating on the face, hands, and axillae. Compensatory sweating worse with sympathicotomy. Satisfaction higher with ramicotomy. Better results with ramicotomy than sympathicotomy regarding hand dryness, how many times a day the patients had to shower or change clothes, intention to undergo the procedure again or recommend it to somebody else, and how bothersome compensatory hyperhidrosis was. T_3_–T_4_ gray ramicotomy had better results than T_3_–T_4_ sympathicotomy, with less compensatory sweating and higher patients' satisfaction.

## Introduction

Hyperhidrosis is a sympathetic nervous system dysfunction in which there is sweating beyond physiological needs^1^. It affects mainly the hands and axillae^2^, making social interaction difficult^3^. Medical treatments are helpful^4,5^, but thoracic sympathectomy (TS) is used in refractory cases^6,7^. Although practised since 1920 ^8^, continuous improvements over the years have led to a progressive reduction in aggressiveness, morbidity, and mortality of TS^6,7^.

Just after the TS patients are pleased as they do not longer sweat in the areas where it was incredibly cumbersome (hands and axillae)^9^, but as time goes by, especially during the summer, they notice sweating in areas that did not sweat before or not to such an extent, such as the abdomen, torso, buttocks, and thighs^10^. This new sweating is known as compensatory hyperhidrosis (CH) and is the fundamental cause of long-term patients' dissatisfaction^11–13^. Another problem, although patients do not complain so often about it^14^, is the over-dryness of the sympathetically denervated area (head/face, hands, and axillae)^15,16^.

Removal of the sympathetic chain is no longer advocated^17,18^. Instead, this chain's surgical section, known as sympathicotomy (SY), is commonplace at different levels^19–21^. However, this leaves the axillae, hands, face, and head with no possibility of sweating and temperature regulation^22,23^. It has to be kept in mind that this area, the upper part of the body, represents 40% of the area through which we control our temperature by heat dissipation through sweating^24^.

Over time the extent of sympathetic chain surgical lesion has been decreased, attempting to reduce the incidence and severity of CH^25,26^. Many agree that the number of sympathetic ganglia injured should include only T_3_ and T_4_^18,27–29^.

Some have tried applying clips instead of irreversibly damaging the nervous tissue^30^. In bothersome CH, these clips can be removed and wait to hope for improvement^31^. Unfortunately, this is not always the case^32,33^.

Attempting further refinement, surgeons lesioned only the white and gray rami communicantes (RC) without injuring the sympathetic chain itself^34^. This surgical technique reduced CH severity, but its incidence remained unaltered^35^.

Some recent studies have tried to lesion only the gray rami communicantes, leaving the white rami communicantes and sympathetic chain intact^36,37^. Both studies showed a further reduction in the incidence and severity of CH, but the number of treated levels seemed excessive (T_2_–T_5_ for Akil et al.^36^ and T_2_–T_4_ for Coveliers et al.^37^).

We hypothesized that the selective lesion of the gray T_3_ and T_4_ rami communicantes would be enough to reduce CH incidence and severity. In addition, this selective sympathetic system lesion should improve the patients' satisfaction and reduce excessive dry hands, gustatory sweating, and cardio-pulmonary disorders (feeling of lack of strength when trying to lift a weight).

## Patients and methods

### Study population

We obtained Ethical and Research Committees' approval, and we performed all methods following the relevant guidelines and regulations following the Helsinki Declaration rules. Clinical registration number CEIm 24-02-2016 (Comité de Ética e Investigación), date of approval 24/02/2016, ClinicalTrials.gov Identifier: NCT04721483, date of registration 22/01/2021.

We conducted this study between May 2016 and September 2019, including forty-two patients distributed in a prospective randomized fashion between two groups (Supplementary figure S1). We calculated the sample size based on previous research and used a block randomization model. We took eight blocks of four and two blocks of five patients with a random distribution of two patients from each group. This project was blind (only the surgeons knew which group the patients belonged to). A team member collected the data, unaware of which specific surgical procedure had undergone each patient. The procedure was a classical T_3_–T_4_ SY in twenty-one patients and a selective T_3_–T_4_ gray RC lesion in the other twenty-one.

Dermatology referred the patients to us once all conservative treatments proved inefficient or the patient refused to continue with them. Unfortunately, seven patients declined to participate in this study.

Data were prospectively collected and analyzed at the end of the study. We focused on the surgical procedures' duration, the length of the hospital stays, the amount and severity of complications, and the sweating and temperature before and after the surgical procedure. We likewise recorded the intraoperative temperature rise in the hands, the quality of life baseline and on follow-ups, as well as the CH. We also registered how many times patients had to shower or change clothes, hand dryness, hand moisturizer, and satisfaction with the results. In addition, we asked patients if they would undergo the same procedure again and recommend it to somebody else. Finally, we measured postoperative B.M.I. changes as they might impact outcomes^38^.

All patients suffered from palmar, axillary, and plantar HH, but the main reason to seek surgical treatment was excessive palmar sweating.

We documented informed consent for each patient, where patients agreed to be allocated randomly to either group and attend follow-up appointments.

The minimum postoperative follow-up required for patients included in our study was 12 months.

A member not participating in the patients' care collected the data.

#### Inclusion criteria

Age 18–60 years, palmar HH with or without axillary HH refractive to conservative treatments or reluctant to continue with them after six months and willing to undergo surgical treatment, Hyperhidrosis Disease Severity Score grade D^39^.

#### Exclusion criteria

Previous thoracic pathology (lung infections, particularly pulmonary empyema, pneumothorax, hemothorax, rib fractures, neoplasms), heart failure, hypothyroidism, tuberculosis, bradycardia (40 pulsations/min), alcoholism, drug addiction, BMI > 30, pregnancy, generalized HH or related to any health disorder, comorbidities or medication intake that induce excessive sweating. We excluded patients with primary facial or plantar HH and those not complying with follow-ups.

### Baseline patients' evaluation

On their first consultation and at each follow-up appointment, patients fulfilled the quality of life questionnaire developed by Amir et al.^40^ (Supplementary table S1). We asked patients not to use any ointment, cream on the skin, or do physical exercise before the evaluation. We recorded baseline sweating and skin temperature at the forehead, palms of hands, axillae, abdomen, thighs, and soles of feet. We placed patients in a closed room for 15 min at 25 °C and 60% humidity (SAIVOD, Shenzhen, China). We collected the sweat with an absorbent pad (DELIPLUS, Mercadona, Paterna, Valencia, Spain). We recorded each absorbent pad's using a precision scale (GRAM FH 6000, Gram Precision S.L., l'Hospitalet de Llobregat, Barcelona, Spain) before and 15 min after being placed in the corresponding anatomical area. The increase in weight corresponded to the sweat produced. We measured the temperature with a laser thermometer (QUIRUMED 574-300, HuBDIC Co., Ltd., Anyang-si, Gyeonggi-do, Korea).

### Surgical procedure

#### Step 1

Patients underwent general anaesthesia with double-lumen endotracheal intubation (VYGON US, Montgomeryville, PA, USA). We inserted intradermal electrodes (MTS-400010, Level Myocardial Temperature Sensor, Smiths Medical ASD, Inc., Rockland, MA 02370 USA) in the thenar eminences. We placed patients in the semi-Fowler position with the arms abducted 45°.

We used a thoracoscope (Storz code 26034AV, Karl Storz Endoscope, Tüttlingen, Germany).

The camera we used was a TELECAM (Storz code 20212030, Karl Storz Endoscope, Tüttlingen, Germany). The TV monitor was a TELE PACK X LED (Karl Storz Endoscope, Tüttlingen, Germany). The optic employed had a bayonet shape, allowing the introduction of pincers, scissors, or a right-angle hook.

After lung collapse, we inserted two portals (THORACIC TROCAR 13 mm/7 cm, Unimax Medical Systems Inc., Hsin Tien, Taipei, Taiwan) at the third intercostal space midaxillary line.

On inserting the portals, the lungs collapsed. Only four patients (4 lungs) required 600–1000 ml of CO_2_ gas insufflation to induce a pneumothorax.

We inspected the pleural cavity and sectioned any adhesions. We undertook a thorough examination to identify the ganglia and the gray RC at T_3_ and T_4_.

#### Step 2

At this moment, patients underwent a T_3_ and T_4_ SY (Step 3A) or a T_3_ and T_4_ gray ramicotomy (RY) (Step 3B).

We opened the parietal pleural 5 mm lateral to the sympathetic chain with an "L" tip monopolar electrode (Storz code 26778 F, Karl Storz Endoscope, Tüttlingen, Germany). Then, we dissected with endoscopic Metzenbaum scissors (34421 MS, Karl Storz Endoscope, Tüttlingen, Germany).

#### *Step 3A: T*_*3*_* and T*_*4*_* sympathicotomy*

The sympathetic chains were interrupted at the third and fourth ribs with harmonic scissors (Harmonic Scissors ACE7® + Laparoscopic Shears HARH45, Ethicon Endo-Surgery, Cincinnati, OH, 45242–2839 USA). We used a harmonic scalpel instead of a monopolar scalpel to achieve a more selective lesion^41,42^. Bleeding from any nearby vessels was controlled by clip application.

#### *Step 3B: T*_*3*_* and T*_*4*_* selective gray ramicotomy*

We only dissected free the T3 and T4 gray RC in this group, leaving the white RC, sympathetic chain, and ganglia untouched.

Once isolated, we sectioned the gray RC with harmonic scissors. We coagulated the pleura over the third and fourth ribs over 2 cm to ensure that we had sectioned all gray RC or Kuntz's nerves. The sympathetic chain was not dissected or elevated from the ribs to avoid damaging it, its ganglia, or the white RC.

### Intraoperative outcome measures

Intraoperative ablation of the sympathetic system was suspected with the change in the pulse oximeter amplitude, indicating increased hand blood circulation reported by other researchers^43^. We used a rise of 0.8 °C or higher to confirm the sympathetic system interruption intraoperatively, as published by other researcher groups^37,44–46^.

#### Step 4: Closure

Once we completed the elected sympathetic nervous system lesion, we inspected the pleural cavity. On inserting a 14-French tube, we applied suction to the pleural cavity to remove all the air and CO_2_, the lung re-expanded, and the anaesthetist used a Valsalva equivalent manoeuvre. Next, we removed the 14-French thoracic tube under positive end-expiratory pressure. A tight subcutaneous purse-string suture ensured airtight sealing, and we closed the skin with an intracutaneous suture. Before discontinuing general anaesthesia, we obtained an anteroposterior plain chest radiograph with the patient in the Fowler position to confirm the absence of any pneumothorax.

### Postoperative hospital stay and care

Postoperatively patients used an incentive spirometer to enhance pulmonary re-expansion. Discharge from the hospital took usually place the day after surgery. We advised patients to return to everyday daily life within 3–7 days and avoid intense physical activity for 15 days.

### Follow-up

One month postoperatively, we measured the sweating and temperature in the same manner and anatomical areas again. In addition, we requested patients complete the same quality of life scale form as fulfilled preoperatively plus a specific postoperative form developed by us (Table 1). These same studies and questionnaires were re-recorded in all follow-ups, at one year postoperatively and in July past the first postoperative year. Afterwards, patients had yearly follow-ups.

**Table 1:**
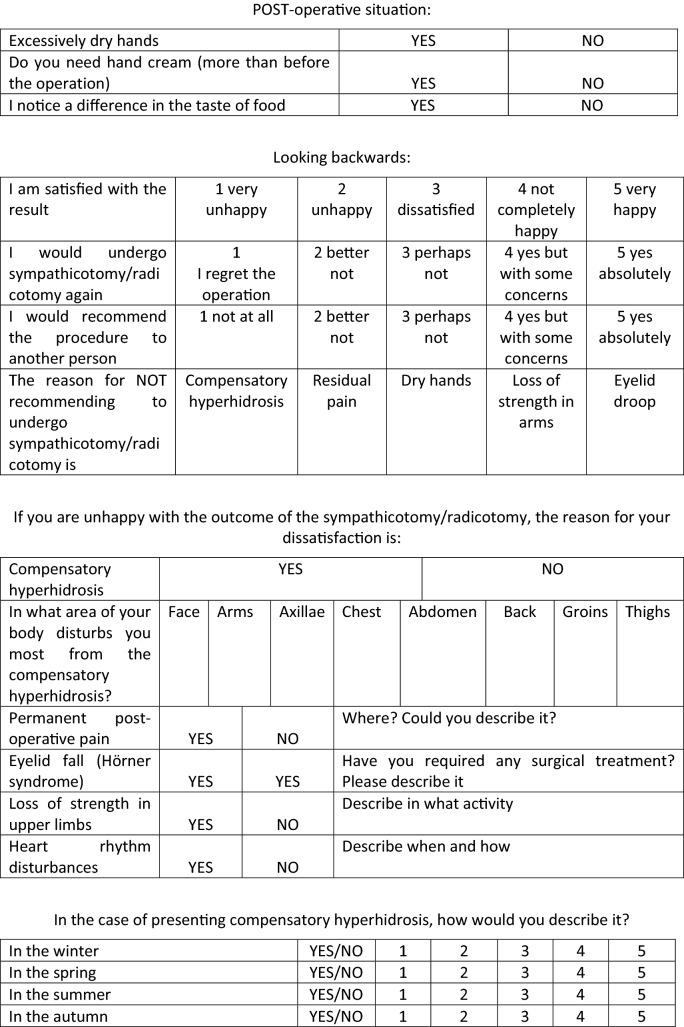

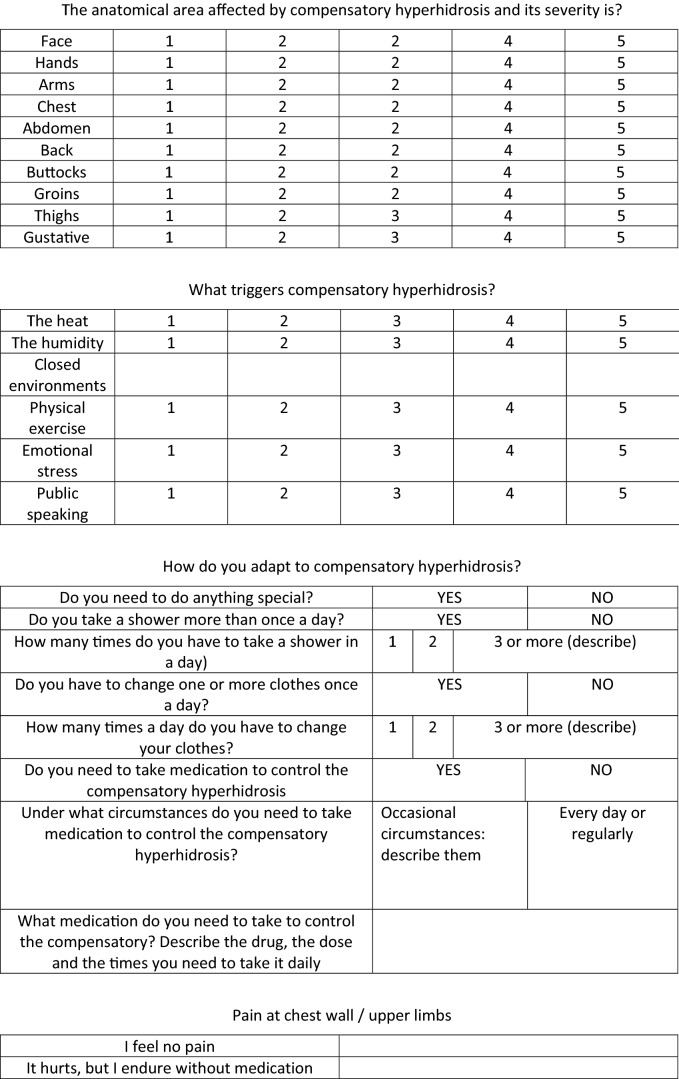

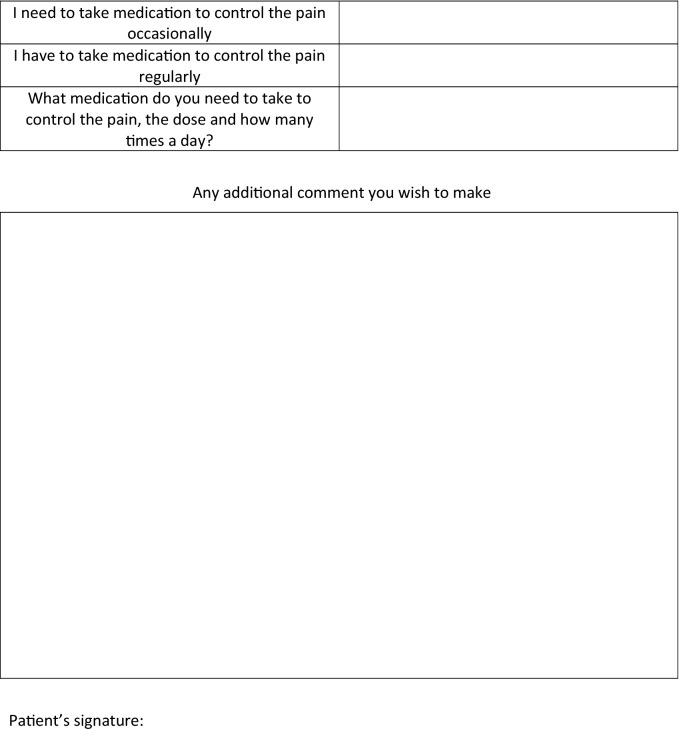
Postoperative quality of life scale form.

A lower score indicates a better quality of life.

### Outcome measures

We evaluated the incidence of pleural adhesions found in the surgical procedure, the time to undertake the operation on each side, the postoperative complications, the hospital stay, and the recurrence rate.

At each follow-up, we recorded sweating and skin temperature at the forehead, palms of hands, axillae, abdomen, thighs, and soles of feet under the same temperature and humidity conditions as in baseline.

Patients graded CH as^35,47–49^ 1-absent, 2-mild, 3-embarrassing or moderate, 4-disabling or severe, and 5-extreme. Grade 2 means minor and intermittent sweating in elevated temperatures or humid environments or during physical activity. Meanwhile, grade 3 represents intense sweating in raised temperatures or high-humidity environments or during intensive physical activities. Lastly, grade 4 is applied when abundant sweating occurs even in mild climates or environments with normal humidity levels or without physical activity, bothering the patient and causing their clothes to get wet so that they need to change them during the day.

The patients graded dryness following the scale proposed by Lee et al. 2004^48^ as 1-excessive dry hand, 2-dry hands, and 3-the same hand sweating as before the operation.

Overall patient satisfaction was measured using a five-point scale with the following grading: very unhappy, unhappy, dissatisfied, not completely happy, or very happy.

Quality of Life Questionnaire^40^, values over 84 indicated a pretty poor quality of life, low from 69 to 84, proper from 36 to 51, and excellent from 20 to 35.

### Primary and secondary endpoints

The primary endpoint was the prospective objective evaluation of CH's incidence and severity in treating primary hyperhidrosis patients with T_3_–T_4_ SY versus T_3_–T_4_ RC in age- and sex-matched controls. In addition, we will present the results as the rate of symptom resolution and the degree of postoperative patient satisfaction.

The secondary endpoints evaluated the incidence of dry hands, gustatory sweating, complication rate, hospital stay, and recurrences of T_3_–T_4_ SY versus T_3_–T_4_ RC.

### Ethical approval

Ethical and Research Committees approval obtained from CEIm Hospital General Universitario de Valencia, 26th of February 2016), ClinicalTrials.gov Identifier: NCT04721483.

### Statistical analysis

We performed it using the statistical software R v3.4.0. (Hornik (2020), “The R FAQ”, https://cran.r-project.org/doc/FAQ/R-FAQ.html#Citing-this-document) on an Excel database (Excel, Microsoft Corporation, Redmond, WA, USA).

The objective was to compare the decrease in the quality of life questionnaire pre and postoperatively for each group.

### Pre-study

When comparing the means of two independent groups, we assumed a two-tailed problem, with an alpha (Type I Error) of 0.005, Power of 0.95 (Type II Error). To obtain the effect size, we assumed that the patient in the RY group suffered a decrease of sixty points on average, and the patients in the SY group suffered a reduction of fifty points on average. We also assumed that both had a similar standard deviation of around nine points so that the effect size was 1.11. With these assumptions, each of the arms needed at least nineteen patients. Adding a 10% patient power loss, at least twenty-one patients in each arm were required.

We conducted the sample size calculation using the G * Power statistical software.

### Post-study

We calculated the mean, standard deviation (SD), median, and interquartile range (IR) for the continuous variables. We compared the groups' continuous variables with the t-test when values were parametric and with the Mann–Whitney U test when they were not. We calculated the percentages for each group and compared them with the chi-square test for the discrete variables. The level of statistical significance used was 5% (*p* value < 0.05).

## Results

We excluded one patient from each group because they did not attend the postoperative follow-ups.

**Figure 1:**
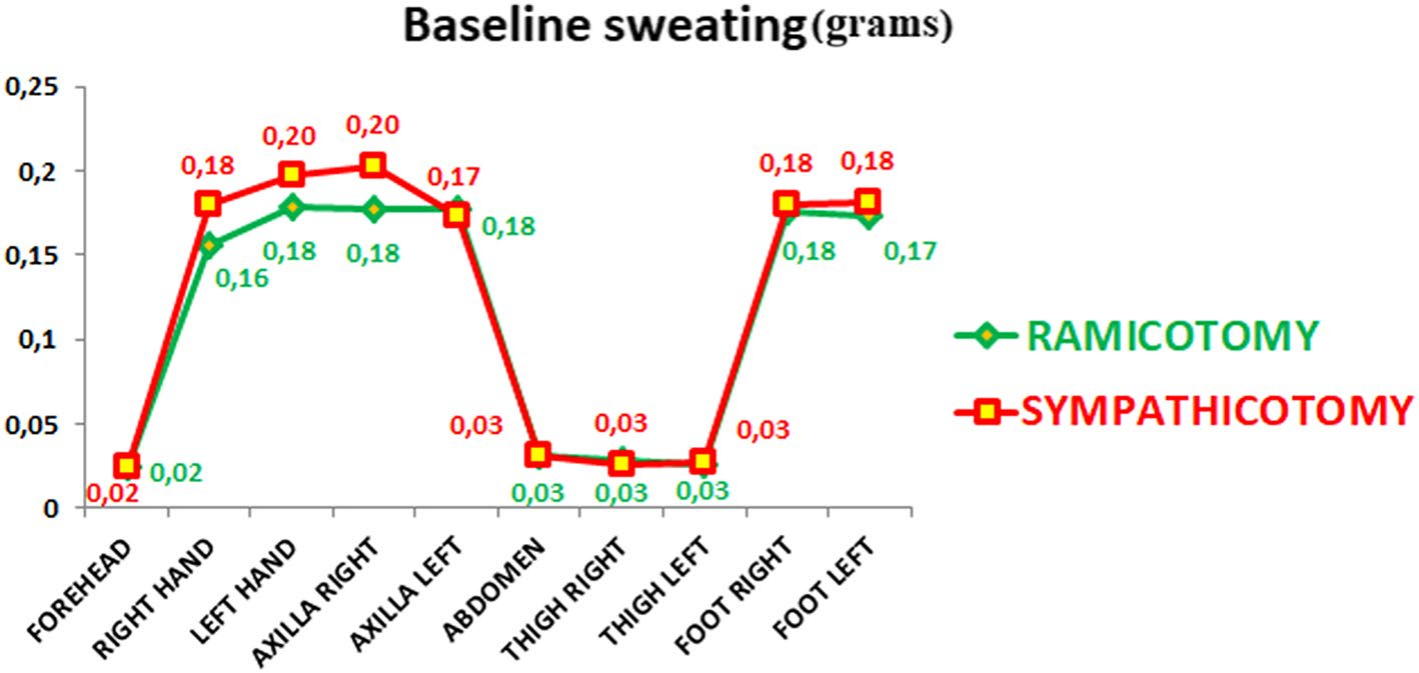
Baseline sweating from the different anatomical areas evaluated.

**Figure 2:**
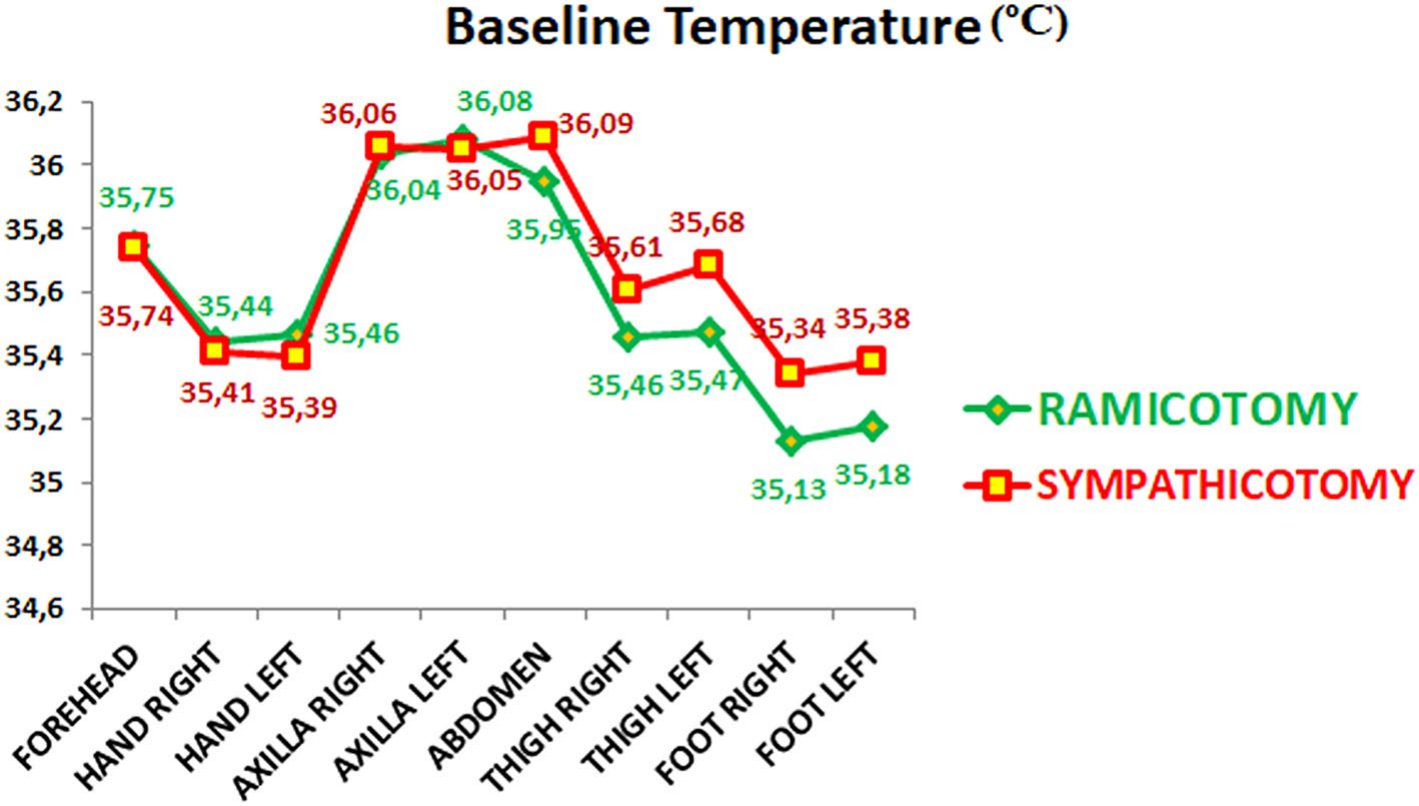
Baseline temperatures from the different anatomical areas evaluated.

Table 2 shows the patient's demographic data and the baseline quality of life according to the group. Preoperatively there were no statistically significant differences between groups based on age, gender, comorbidities, smoking, BMI, quality of life, sweating (Fig. 1, Supplementary table S4), and temperature (Fig. 2, Supplementary table S5).

**Table 2:** Patients’ demographic data and baseline quality of life according to the group. The higher the figure, the worse is the quality of life.

Variable	Ramicotomy	Sympathicotomy	*p* value
	20 (50%)	20 (50%)	
***Age (years)***			0.949
Mean (SD)	29.80 (8.41)	30.00 (10.90)	
Median (IR)	30.00 (23.00–35.50)	30.00 (21.75–36.50)	
***Sex***			0.514
Men	9 (45.00%)	6 (30.00%)	
Women	11 (55.00%)	14 (70.00%)	
***Height (centimetres)***			0.084
Mean (SD)	169.20 (9.37)	164.35 (7.85)	
Median (IR)	168.50 (160.75–175.75)	165.00 (158.50–170.00)	
***Weight (kilograms)***			0.476
Mean (SD)	67.70 (11.69)	65.00 (12.04)	
Median (IR)	66.00 (58.75–79.00)	62.00 (56.75–72.75)	
***BMI***			0.576
Mean (SD)	23.49 (2.24)	24.12 (4.50)	
Median (IR)	23.56 (22.31–25.47)	23.21 (20.31–27.03)	
***Smoker***			0.236
No	14 (70.00%)	18 (90.00%)	
Yes	6 (30.00%)	2 (10.00%)	
***Family hyperhidrosis***			0.527
No	11 (55.00%)	8 (40.00%)	
Yes	9 (45.00%)	12 (60.00%)	
***Age start hyperhidrosis***			0.160
Mean (SD)	10.20 (7.11)	7.45 (4.75)	
Median (IR)	8.50 (6.00–13.00)	6.00 (5.00–8.75)	
***Previous treatments***			0.177
No treatment	9 (45.00%)	4 (20.00%)	
***Quality life baseline***			0.772
Mean (SD)	87.15 (6.26)	87.70 (5.63)	
Median (IR)	88.00 (83.25–91.25)	88.50 (86.00–91.00)

Only one patient belonging to the SY group had pleural adhesions that we lysed uneventfully. No surgery required conversion to an open thoracotomy. We could control all intraoperative haemorrhages endoscopically.

Intraoperatively thenar eminence temperature rose more in the SY than in the RY (right hand 0.92 (SD: 0.51), IR: 0.50–1.20 vs. 0.51 (SD: 0.19), IR: 0.48–0.70, left hand 0.88 (SD: 0.41), IR: 0.50–1.02 vs. 0.62 (SD: 0.21, IR: 0.48–0.80)) (Supplementary table S6). The postoperative stay was longer for the SY group (2.05, SD: 1.82 vs. 1.15, SD: 0.37) due to one case of hemothorax and another to pneumothorax requiring inserting a thoracic drain. The bleeding was controlled endoscopically during the surgical procedure, but a drain was inserted to ease blood removal postoperatively and shorten postoperative stay. We had no mortality and no Horner's syndromes. The surgical complications only lengthened the hospital stay.

The mean follow-up is 33 months (Range: 22–44).

**Figure 3:**
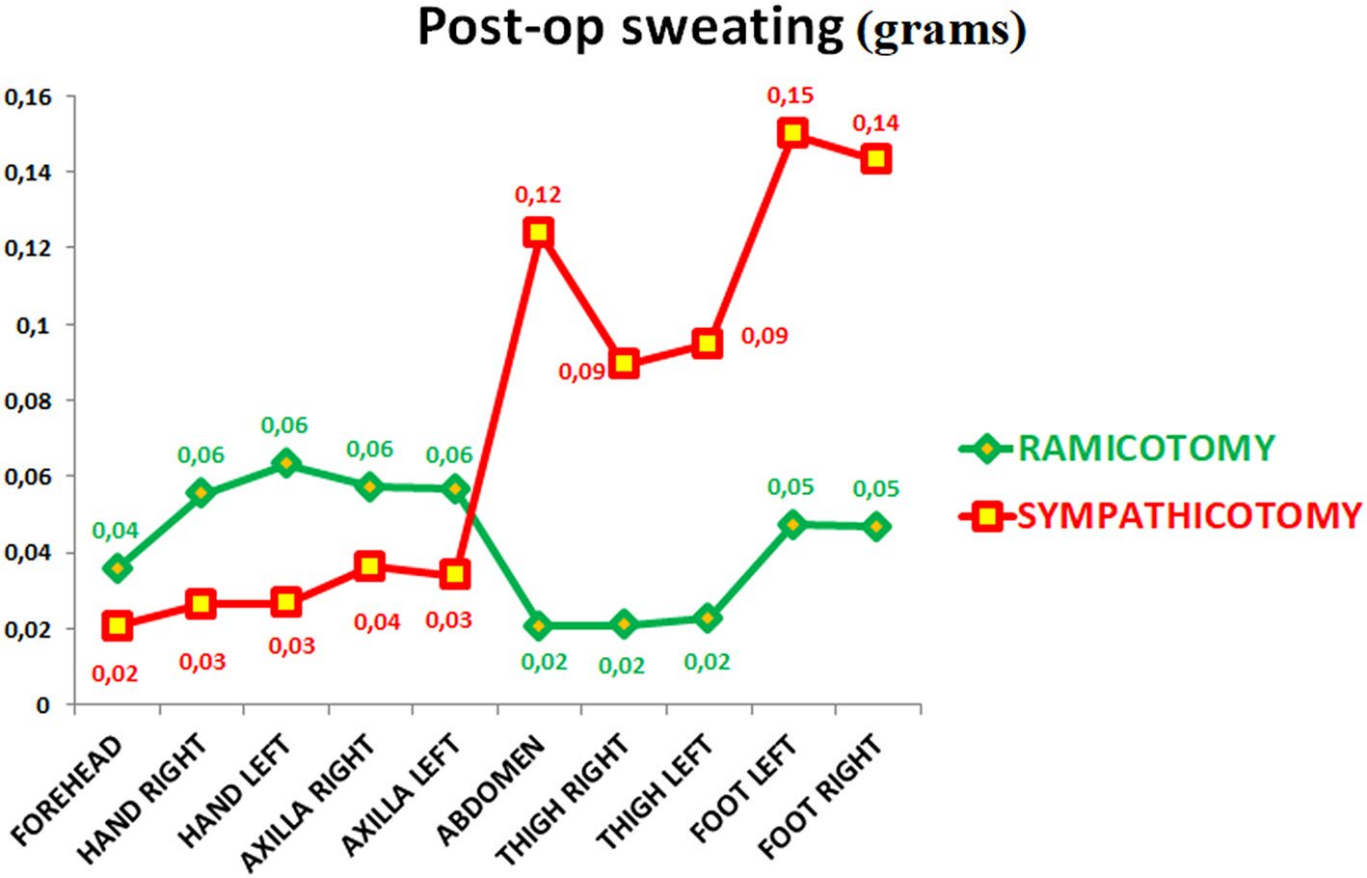
Postoperative sweating in different anatomical areas. Please notice some residual sweating in the partially sympathetically denervated areas in the RY, while there is almost none in the sympathicotomy. In addition, there is an increase in sweating in the abdomen, thighs, and feet in this last group that does not happen in the first group.

**Figure 4:**
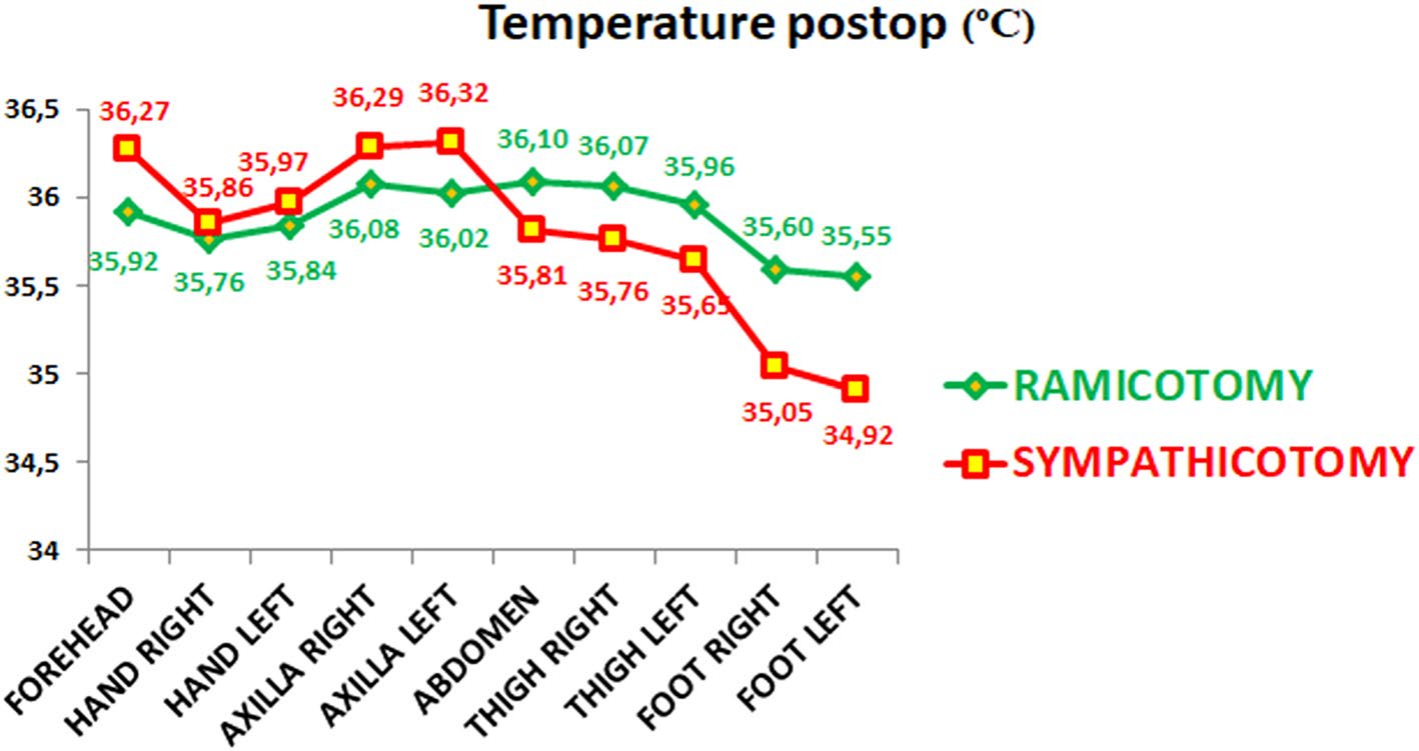
Postoperative temperature of the different anatomical areas. We see a more significant drop in the abdomen, thighs, and feet in the sympathicotomy group, attesting that they suffer from a more substantial CH. Also, notice that the temperature rises more in the forehead, hands, and axillae, showing that the sympathetic denervation is more prominent in the sympathicotomy group.

The following data were recorded in July, coinciding with the warmest temperatures and maximum humidity. Analyzing the postoperative quality of life scale, patients undergoing SY reported worse results (49.05 (SD: 15.66), IR: 35.50–63.00 vs. 24.30 (SD: 6.02), IR: 19.75–27.25) (Table 3). A higher figure in the quality of life scale means a smaller improvement postoperatively. Patients in the SY group sweated less in the hands, axillae, and forehead but much more in the abdomen, thighs, and feet (Fig. 3, Supplementary table S7). SY patients had a more significant postoperative temperature rise in the forehead, with a colder temperature in the abdomen, thighs, and soles of feet than those in the RY group (Fig. 4, Supplementary table S8).

**Table 3:** Postoperative quality of life and sweating changes in different anatomical areas during July.

Variable	Ramicotomy	Sympathicotomy	*p* value
	20 (50%)	20 (50%)	
***Quality life POSTOP year***			** < 0.001**
Mean (SD)	24.30 (6.02)	49.05 (15.66)	
Median (IR)	23.50 (19.75–27.25)	53.50 (35.50–63.00)	
***BMI postop year***			
Mean (SD)	23.41 (2.14)	24.41 (3.99)	0.331
Median (IR)	23.33 (22.10–24.77)	23.52 (21.82–26.17)	
***Shower more once a day***			** < 0.001**
No	20 (100.00%)	7 (35.00%)	
Yes	0 (0.00%)	13 (65.00%)	
***Change dress times days***			** < 0.001**
1	20 (100.00%)	6 (30.00%)	
2	0 (0.00%)	8 (40.00%)	
3	0 (0.00%)	6 (30.00%)	
***Hands too dry***			**0.013**
NO	20 (100.00%)	13 (65.00%)	
YES	0 (0.00%)	7 (35.00%)	
***Need hand cream***			**0.002**
NO	20 (100.00%)	11 (55.00%)	
YES	0 (0.00%)	9 (45.00%)	
***Happy result***			** < 0.001**
1 very unhappy	0 (0.00%)	1 (5.00%)	
2 unhappy	0 (0.00%)	2 (10.00%)	
3 dissatisfied	0 (0.00%)	4 (20.00%)	
4 not completely happy	3 (15.00%)	11 (55.00%)	
5 very happy	17 (85.00%)	2 (10.00%)	
***WOULD Would you have the operation again YOU HAVE THE OPERATION_AGAIN***			**0.002**
1 I regret the operation	0 (0.00%)	1 (5.00%)	
2 better not	0 (0.00%)	2 (10.00%)	
3 perhaps not	0 (0.00%)	7 (35.00%)	
4 yes, but with some concerns	3 (15.00%)	5 (25.00%)	
5 yes, absolutely	17 (85.00%)	5 (25.00%)	
***RRecommend operation to somebody elseECOMEND_OPERATION TO_SOMEBODY_ELSE***			** < 0.001**
1 not at all	0 (0.00%)	1 (5.00%)	
2 better not	0 (0.00%)	1 (5.00%)	
3 perhaps not	0 (0.00%)	4 (20.00%)	
4 yes but with some concerns	0 (0.00%)	7 (35.00%)	
5 yes, absolutely	20 (100.00%)	7 (35.00%)	
***Compensatory hyperhidrosisCOMPENSATORY_HYPERHIDROSIS***			** < 0.001**
1 No	17 (85.00%)	1 (5.00%)	
2 intermittent or minor	3 (15.00%)	0 (0.00%)	
3 embarrassing	0 (0.00%)	2 (10.00%)	
4 disabling	0 (0.00%)	4 (20.00%)	
5 severe	0 (0.00%)	13 (65.00%)	

Patients undergoing SY reported worse results than RC ones. The higher figure in the quality of life scale means that there was minor improvement postoperatively. Analyzing the BMI (body mass index at the time of follow-up in July), we found no statistically significant differences between the groups. The CH and its consequences were milder in the gray rami communicantes RY than in the SY group.

significance is for p <0.05

We could not attribute the postoperative differences observed in sweating, temperature, and CH to BMI changes as we saw no statistically significant differences between the groups (SY 24.41, SD: 3.99 vs. RY 23.41, SD: 2.14) (Table 3).

Patients completed a postoperative form asking about the CH (Table 1), where they experienced it, where it bothered most, its severity, and the adopted adaptive measures. Again, results were better for the RY than for the SY group (Table 3).

The preoperative and postoperative July past one-year follow-up data was compared (Supplementary table S2). Although all patients improved, this improvement was more significant in the RY group. In addition, the temperature changes were milder for the RY group than the SY patients (Supplementary table S3). These temperature changes attest to a milder sympathetic lesion for the first than for the second group.

Patients have reported no recurrences at this time, with a maximum follow-up of 4 years.

## Discussion

Ever since the introduction of TS in the treatment of palmar and axillary HH, there has been a continuous quest to find a way to reduce its most unpleasant side effect: CH^24^. Following the idea of minimizing the surgically induced damage to the sympathetic chain Wittmoser^34^ introduced in 1992 RY's technique. It entailed the selective lesion of the rami communicantes, both white and gray, from T_2_ to T_4_ ± T_5_. The procedure ended with the sympathetic chain's elevation from its bed to ascertain that no rami communicantes, either gray or white, were left intact. This surgical technique was not as selective as expected because it damaged the sympathetic input for the lungs and heart and the head and face^34^. Wittmoser described this surgical technique but did not undertake any clinical trial and never published any results. Gossot^35^ in 1997 reported his results in 62 patients in whom he lesioned the T_2_ to T_4_ ± T_5_ gray and the white rami communicantes with the elevation of the sympathetic chain. He compared this technique with the SY of the same ganglia in fifty-four patients. He found that CH's incidence was the same in both groups, but the severity was less in the RY than in the SY group. However, the recurrence rate was more prominent in the first than in the second group (5% RY vs. 0% SY). This increase in the recurrence rate was a deterrent for spreading this surgical technique of grey and white rami communicantes lesion. Nevertheless, this study showed that lesser sympathetic denervation meant that the hands' palms could retain residual sweating^35^. Other researchers have confirmed these results^48,50^ (Table 4).

**Table 4:** Summary of the studies on endoscopic thoracic RY over the years.

Author	Year	No. pts SY/RY	Surgical technique SY/RY	Success rate (%)	% CH	% GS	% Dry hands	PHH	% RH	Follow-up post-op	Satisfaction rate (%)
Akil et al	2018	0/51	–/T_2_–T_5_ gray rami lesion Sch NOT lifted	–/100	–/0	ND	ND	ND	ND	4 mo, phone or email 12 ± 2.5 months	–/100
Coveliers et al	2013	0/55	–/T_2_–T_4_ gray rami lesion Sch NOT lifted	–/96	–/7.2	–/1.8	ND	ND	ND	24 mo (3–36 months)	ND
Hwang et al	2013	46/43	T_3_ SY/T_3_–T_4_ gray + white rami lesion Sch lifted	97.8/83.7	37.2/10.9severe in 14/8.7	ND	82.6/25.6	15.2/58.5	ND	At 1 month + phone 1-year	79.1/91.3
Lee et al	2004	64/83	T_2_ SY/T_3_ Gray + white rami lesion Sch lifted	93.8/69.9	43.3/15.5	ND	6.2/18.1	6.25/30.1	0/8.8	Phone 9.7 ± 1.3 vs 6.6 ± 3.7 months	78.1/68.6
Kim et al	2004	22/22	T_2_ clip/T_2_ gray + white rami lesion Sch lifted	ND	75.5/36.4	4.4/0	22.7/13.6	0/18.3	ND	Phone 20.1 ± 7.7 vs 10.8 ± 2.0 mo	77.3/63.6
Lee et al	2003	40/68	T_3_ clip/T_3_ gray + white rami lesion Sch lifted	90/67.6	94.1/67.4	ND	ND	8.9/23.5	ND	Phone 6.6 ± 3.7 vs 28.2 ± 6.2 mo	82.5/67.6
Cho et al	2003	13/13	T_3_ clip/T_3_ RY technique ND	ND	92.9/54.5	ND	SY only % 1.4 out of 4	RY only % 2 out of 4	6.7/21.4	ND	5.5/6.5 out of 10
Cheng et al	2001	34/13	T_2_–T_3_ SY/T_2_–T_3_ gray + white lesion Sch lifted	ND	82/23.07	ND	ND	ND	0/15.38	Mean 9 mo	ND
Gossot et al	1997	54/62	T_2_–T_4_ ± T_5_ SY/T_2_–T_4_ ± T_5_ gray + white rami lesion Sch lifted	100/100	50/21	ND	ND	ND	0/5	Mean 11 mo	ND
Wittmoser et al	1992	–/–	T_2_–T_4_ ± T_5_ gray + white rami lesion	NA	NA	NA	NA	NA	NA	NA	NA

Sympathicotomy (SY). Ramicotomy (RY). Sch (Sch) Compensatory sweating (CH). Gustatory sweating (GS). Persistent hand humidity (PHH). Recurrence hyperhidrosis (RH). Not described (ND). Phone (follow-up performed through a telephone questionnaire).

In the following years, more studies compared the SY with the RY, confirming that RY has a lower incidence of CH^15,47,48,50–52^, with less dry hands^47,48,50,52^ but with some recurrences^15,48,50^. The technique used to perform the RY kept being the section of both the gray and white rami communicantes with the sympathetic chain's elevation. On further analysis of this group of publications, we realize that the number of levels treated is not uniform. Two of them include the rami communicantes of the T_2_ ganglion^15,47^. Nowadays, we know that lesioning this ganglion should be avoided as it correlates with a more severe CH^14,18,26,53–55^. In three other studies, only the rami communicantes of the T_3_ ganglion are sectioned^48,50,51^. These studies report the most significant recurrence rate (8.8^48^ and 21.4%^50^). In one study, researchers reported T_2_ and T_3_ gray and white rami communicantes lesions with the sympathetic chain's elevation^15^. The researchers in this group say an incidence of CH of 23.07% and a recurrence rate of 15.38%. Lesion of T_3_ and T_4_ gray and white rami communicantes had an incidence of CH of 10.9% and no recurrence. In conclusion, the recommendation is to avoid T_2_ lesions and damage only T_3_ and T_4_.

In a step forward, Coveliers et al.^37^ lesioned only the T_2_–T_4_ gray rami communicantes not touching the sympathetic chain nor the white rami communicantes. The incidence of CH dropped to 7.2%, and they do not report any recurrence. Akil et al.^36^ reported the lesion of the T_2_–T_5_ gray rami communicantes with no CH and no recurrences. HH's rate of control ranges from 96%^37^ to 100%^36^. Both studies entail a more extensive lesion, including T_2_^36,37^ and one of those studies, T_5_^36^. There is an agreement in many previous reports to avoid lesioning T_2_ to reduce the incidence and severity of CH^14,18,26,53,55,56^.

Table 4 summarizes the results of all the research groups involved over the years with RY.

The first problem with analyzing all these research groups is that they report CH's incidence and severity based on the patients' subjective report^15,35–37,47,48,51,52^. Some researchers report discrepancies between subjective perception and the objective measurement of hyperhidrosis and CH^57,58^. Moreover, different patients react differently to the same degree of residual or new sweating^59,60^. The oldest method to measure sweat production, the Quinizarin sweat test, is inaccurate ^61,62^. Gravimetry is more objective but still far from ideal. It entails giving a pre-weighted tissue, cotton swab, or piece of filter paper to the patient and asking them to wipe the area under investigation for 1 min in a room with temperature 24–25 °C and humidity 15–17%. Then, the investigator weighs the tissue, cotton swab, or piece of filter paper again, and the increase in weight gives the amount of sweat in milligrams^63–65^. This technique of sweat measurement did not seem accurate enough for our study. The most objective method is the VapoMeter® closed-chamber device (Delfin Technologies Ltd, Kuopio, Finland)^66,67^. The problem is that it only analyzes a tiny skin area (about 2.5 cm in diameter) at a time. Our study measured sweat production in milligrams of water recovered from an absorbent pad and temperature changes measured in degrees Celsius.

The second drawback is that in all the studies, the researchers did the follow-up through telephone^36,47,48,51,52^ or email^36^ questionnaires, which could be insufficient for objective assessment of the percentage and severity of CH. For this reason, our study exclusively used face-to-to-face follow-ups in our out-patients Department.

In our study, all the SY patients and the RY groups got complete relief from the palmar HH. The T_3_–T_4_ gray rami communicantes RY has shown much better results than SY levels concerning CH, dry hands, and patient satisfaction. Although the surgical time is somewhat longer in RY than in the SY group, this extra time seems warranted to achieve better results. No mortalities or severe complications happened in any group, besides one case of pneumothorax and another of hemothorax. Hence the quality of the result must guide our decision when using one surgical technique or the other. The lesion of T_2_ and T_5_ gray rami communicantes, practised by other researchers^36,37^, does not seem justified^18,28,29^.

An additional advantage is that the gray rami communicantes RY leaves some residual sweating in the hands. Former studies involving gray and white rami communicantes lesions already reported this residual hand moisture^47,48,51,52^. However, researchers make no comments on this particular issue in the only two reports involving only the gray rami communicantes lesion ^36,37^. In our study, the need for a hand cream to combat postoperative hand dryness is present in 45% of the SY group patients and none of the RY patients (Table 3). This need for hand cream shows that postoperatively the hands are less dry in the RY than in the SY group.

## Limitations

Our study's limitations are the reduced number of patients in each group and the need to gather more long-term data, particularly on possible H.H. recurrences.

## Strengths

It is a prospective randomized study. A third party recorded the data, and the follow-ups were done by attending patients face-to-face and not through telephone or email questionnaires.

## Conclusions

Compared with T_3_–T_4_ SY, T_3_–T_4_ gray RY shows better results. In addition, patients suffer from less CH and manifest greater satisfaction. Therefore, a more extensive RY does not seem necessary.

## Supplementary Information

Supplementary Information 1.

Supplementary Information 2.

Supplementary Information 3.

Supplementary Information 4.

Supplementary Information 5.

Supplementary Information 6.

Supplementary Information 7.

Supplementary Information 8.

Supplementary Information 9.

Supplementary Information 10.
