# Supplementary figures and images for: Selective T3–T4 sympathicotomy versus gray ramicotomy on outcome and quality of life in hyperhidrosis patients: a randomized clinical trial
Source: Sci Rep. 2021 Sep 2;11:17628. doi: 10.1038/s41598-021-96972-7 (PMC8413289; doi:10.1038/s41598-021-96972-7)

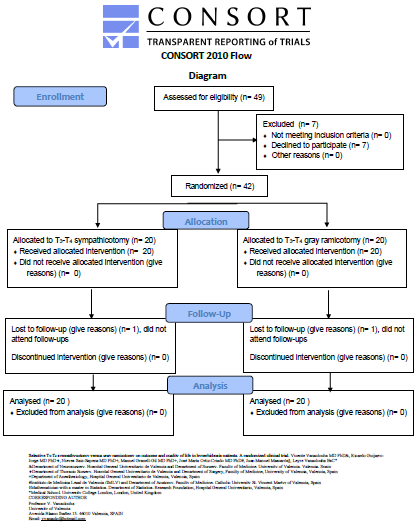

Supplement: Supplementary file 1 — Supplementary Information 1. [file 41598_2021_96972_MOESM1_ESM.tif]
