# Supplementary material for: Selective T3–T4 sympathicotomy versus gray ramicotomy on outcome and quality of life in hyperhidrosis patients: a randomized clinical trial
Source: Sci Rep. 2021 Sep 2;11:17628. doi: 10.1038/s41598-021-96972-7 (PMC8413289; doi:10.1038/s41598-021-96972-7)
Supplement: Supplementary file 2 — Supplementary Information 2. [file 41598_2021_96972_MOESM2_ESM.docx]

**SELECTIVE T_3_-T_4_ SYMPATHICOTOMY VERSUS GRAY RAMICOTOMY ON OUTCOME AND QUALITY OF LIFE IN HYPERHIDROSIS PATIENTS: A RANDOMIZED CLINICAL TRIAL.**

**SUPPLEMENTARY MATERIAL**

**SUPPLEMENTARY FIGURES**

**Figure S1:** patients' flow chart.

**SUPPLEMENTARY TABLES**

**Table S1:** Preoperative quality of life scale form. A higher score indicates a lower quality of life.

**Table S2:** Preoperative and postoperative July past one-year follow-up quality of life and sweating data compared. A high value implies worsening, and a smaller value signifies an improvement. In general, almost all patients improved, but there is a tendency for superior improvement in the rami communicantes RY group.

**Table S3**: Preoperative and postoperative July past one-year follow-up temperature data compared. A high value implies worsening, and a smaller value signifies an improvement. The temperature changes are milder for the gray rami communicantes group compared to the SY patients. These lesser temperature changes attest that the degree of sympathetic system lesion is gentler for the first than for the second group.

**Table S4:** Baseline quality of life and sweating according to the group. We measured the sweat in milligrams of water.

**Table S5:** Baseline temperature per anatomical area according to the group measured in degrees Celsius.

**Table S6:** Temperature measured intraoperatively in the thenar eminence rose more in the SY than in the gray rami communicantes lesion (RC). Postoperative stay length is longer for the SY group because they had one pneumothorax and another hemothorax.

**Table S7:** Postoperative sweating changes in different anatomical areas during July. Patients undergoing SY reported worse results than RC ones. Patients in the SY group sweated less in the hands, axillae, and forehead but much more than they used to in the abdomen, thighs, and feet. This extra sweat in areas where they did not sweat so much means that they suffer from worse CH.

**Table S8**: Postoperative temperature changes in different anatomical areas. SY patients had a more significant rise in the forehead, with a colder temperature in the abdomen, thighs, and soles of feet than in the RY gray rami communicantes group. These temperature changes indicate that SY induces a more prominent sympathetic system lesion and that the gray rami communicantes RY causes a more selective lesion with fewer side effects.
