# Supplementary material for: Selective T3–T4 sympathicotomy versus gray ramicotomy on outcome and quality of life in hyperhidrosis patients: a randomized clinical trial
Source: Sci Rep. 2021 Sep 2;11:17628. doi: 10.1038/s41598-021-96972-7 (PMC8413289; doi:10.1038/s41598-021-96972-7)
Supplement: Supplementary file 3 — Supplementary Information 3. [file 41598_2021_96972_MOESM3_ESM.docx]

| Patient: | | Date: |
| --- | --- | --- |
| Date sympathicotomy/ramicotomy: | Medical Record Number | |

Generally speaking, how would you rate your quality of life at THIS time:

| 1: excellent | 2: very good | 3: good | 4: poor | 5: very poor |
| --- | --- | --- | --- | --- |

Using the same scale above (1-5), how would you rate the following activities:

| Writing | 1 | 2 | 3 | 4 | 5 |
| --- | --- | --- | --- | --- | --- |
| Hand labor | 1 | 2 | 3 | 4 | 5 |
| Spare time | 1 | 2 | 3 | 4 | 5 |
| Sport activities | 1 | 2 | 3 | 4 | 5 |
| Shake hands | 1 | 2 | 3 | 4 | 5 |
| Social life | 1 | 2 | 3 | 4 | 5 |
| Pick up objects | 1 | 2 | 3 | 4 | 5 |
| Dance | 1 | 2 | 3 | 4 | 5 |

Personal area: with your partner, how would you rate your quality of life?

| Holding hands | 1 | 2 | 3 | 4 | 5 |
| --- | --- | --- | --- | --- | --- |
| Intimate touch | 1 | 2 | 3 | 4 | 5 |
| Intimate life | 1 | 2 | 3 | 4 | 5 |

Emotional area, own or with others: how would you rate how excessive sweating affects you

| It affects me | 1 | 2 | 3 | 4 | 5 |
| --- | --- | --- | --- | --- | --- |
| People reject me | 1 | 2 | 3 | 4 | 5 |

Under particular circumstances: how would you rate your quality of life?

| In a closed or hot environment | 1 | 2 | 3 | 4 | 5 |
| --- | --- | --- | --- | --- | --- |
| When worried or tense | 1 | 2 | 3 | 4 | 5 |
| When you think of a problem | 1 | 2 | 3 | 4 | 5 |
| Before an exam, interview, appointment or public speaking | 1 | 2 | 3 | 4 | 5 |
| When you walk barefoot or with sandals | 1 | 2 | 3 | 4 | 5 |
| When wearing coloured clothes | 1 | 2 | 3 | 4 | 5 |
| When you have problems at school or work | 1 | 2 | 3 | 4 | 5 |

| SUBTOTAL |  |  |  |  |  | |
| --- | --- | --- | --- | --- | --- | --- |
| **TOTAL** | | | | | |  |

Patient’s signature:

| BMI |  | | Smoking (cigarettes/day) |  |
| --- | --- | --- | --- | --- |
| Affected relatives | YES | NO | Who in the family is affected? |  |
| Palmar hyperhidrosis | Axillary hyperhidrosis | | Plantar hyperhidrosis | Facial hyperhidrosis |
| YES/NO | YES/NO | | YES/NO | YES/NO |
| Previous lung diseases (YES/NO) | | |  | |
| Result tests pre-op respiratory function | | |  | |
| Previous treatments (YES/NO) | | |  | |
| Which previous treatments? | | |  | |
| How long did you receive non-surgical treatments? | | |  | |
| Profession | | |  | |

Since when do you have hyperhidrosis?

| Since childhood |  |
| --- | --- |
| Since adolescence |  |
| Since youth |  |
| From what pathology? Please specify |  |

Any additional comment you wish to make

|  |
| --- |

Patient’s signature:

**Table S1:** Preoperative quality of life scale form. A higher score indicates a lower quality of life.

Selective T_3_-T_4_ sympathicotomy versus gray ramicotomy on outcome and quality of life in hyperhidrosis patients: a randomized clinical trial. Vicente Vanaclocha MD PhD&, Ricardo Guijarro-Jorge MD PhD♦, Nieves Saiz-Sapena MD PhD+, Manuel Granell-Gil MD PhD+, José María Ortiz-Criado MD PhD#, Juan Manuel Mascarós§, Leyre Vanaclocha BsC*

&Department of Neurosurgery, Hospital General Universitario de Valencia and Department of Surgery, Faculty of Medicine, University of Valencia, Valencia, Spain

♦Department of Thoracic Surgery, Hospital General Universitario de Valencia and Department of Surgery, Faculty of Medicine, University of Valencia, Valencia, Spain

+Department of Anesthesiology, Hospital General Universitario de Valencia, Valencia, Spain

#Instituto de Medicina Legal de Valencia (IMLV) and Department of Anatomy, Faculty of Medicine, Catholic University St. Vincent Martyr of Valencia, Spain

§Mathematician with a master in Statistics, Department of Statistics, Research Foundation, Hospital General Universitario, Valencia, Spain

*Medical School, University College London, London, United Kingdom

CORRESPONDING AUTHOR

Professor V. Vanaclocha

University of Valencia

Avenida Blasco Ibañez 15, 46010 Valencia, SPAIN

Email: [vivava@uv.es](mailto:vivava@uv.es)
