# Supplementary material for: Selective T3–T4 sympathicotomy versus gray ramicotomy on outcome and quality of life in hyperhidrosis patients: a randomized clinical trial
Source: Sci Rep. 2021 Sep 2;11:17628. doi: 10.1038/s41598-021-96972-7 (PMC8413289; doi:10.1038/s41598-021-96972-7)
Supplement: Supplementary file 4 — Supplementary Information 4. [file 41598_2021_96972_MOESM4_ESM.docx]

| **Variable** | **Ramicotomy** | **Sympathicotomy** | **P-value** |
| --- | --- | --- | --- |
|  | 20 (50%) | 20 (50%) |  |
| ***Quality life difference*** |  |  | **<0.001** |
| Mean (SD) | -62.85 (7.36) | -38.65 (15.42) |  |
| Median (IR) | -63.00 (-69.00–56.75) | -37.00 (-50.25–26.75) |  |
| ***Forehead difference sweat*** |  |  | **0.011** |
| Mean (SD) | 0.01 (0.01) | 0.00 (0.02) |  |
| Median (IR) | 0.01 (0.00-0.02) | 0.00 (-0.01-0.00) |  |
| ***Right-hand difference sweat*** |  |  | **0.002** |
| Mean (SD) | -0.10 (0.05) | -0.15 (0.05) |  |
| Median (IR) | -0.09 (-0.12–0.08) | -0.15 (-0.19–0.12) |  |
| ***Left-hand difference sweat*** |  |  | **0.012** |
| Mean (SD) | -0.12 (0.06) | -0.17 (0.07) |  |
| Median (IR) | -0.09 (-0.17–0.07) | -0.17 (-0.21–0.12) |  |
| ***Left Axilla difference sweat*** |  |  | 0.445 |
| Mean (SD) | -0.12 (0.06) | -0.14 (0.08) |  |
| Median (IR) | -0.11 (-0.13–0.08) | -0.12 (-0.15–0.09) |  |
| ***Right Axilla difference sweat*** |  |  | 0.093 |
| Mean (SD) | -0.12 (0.05) | -0.17 (0.11) |  |
| Median (IR) | -0.11 (-0.15–0.08) | -0.12 (-0.22–0.10) |  |
| ***Abdomen difference sweat*** |  |  | **0.006** |
| Mean (SD) | -0.01 (0.01) | 0.09 (0.15) |  |
| Median (IR) | -0.01 (-0.02-0.00) | 0.06 (0.04-0.09) |  |
| ***Right thigh difference sweat*** |  |  | **<0.001** |
| Mean (SD) | -0.01 (0.01) | 0.06 (0.03) |  |
| Median (IR) | -0.01 (-0.02-0.00) | 0.06 (0.05-0.08) |  |
| ***Left thigh difference sweat*** |  |  | **<0.001** |
| Mean (SD) | 0.00 (0.01) | 0.07 (0.03) |  |
| Median (IR) | 0.00 (-0.01-0.00) | 0.06 (0.05-0.08) |  |
| ***Right Foot difference sweat*** |  |  | **<0.001** |
| Mean (SD) | -0.13 (0.07) | -0.04 (0.06) |  |
| Median (IR) | -0.12 (-0.16–0.09) | -0.04 (-0.07-0.00) |  |
| ***Left Foot difference sweat*** |  |  | **<0.001** |
| Mean (SD) | -0.13 (0.06) | -0.03 (0.07) |  |
| Median (IR) | -0.12 (-0.15–0.09) | -0.02 (-0.07–0.01) |  |

**Table S2:** Preoperative and postoperative July past one-year follow-up quality of life and sweating data compared. A high value implies worsening, and a smaller value signifies an improvement. In general, almost all patients improved, but there is a tendency for superior improvement in the rami communicantes RY group.

Selective T_3_-T_4_ sympathicotomy versus gray ramicotomy on outcome and quality of life in hyperhidrosis patients: a randomized clinical trial. Vicente Vanaclocha MD PhD&, Ricardo Guijarro-Jorge MD PhD♦, Nieves Saiz-Sapena MD PhD+, Manuel Granell-Gil MD PhD+, José María Ortiz-Criado MD PhD#, Juan Manuel Mascarós§, Leyre Vanaclocha BsC*

&Department of Neurosurgery, Hospital General Universitario de Valencia and Department of Surgery, Faculty of Medicine, University of Valencia, Valencia, Spain

♦Department of Thoracic Surgery, Hospital General Universitario de Valencia and Department of Surgery, Faculty of Medicine, University of Valencia, Valencia, Spain

+Department of Anesthesiology, Hospital General Universitario de Valencia, Valencia, Spain

#Instituto de Medicina Legal de Valencia (IMLV) and Department of Anatomy, Faculty of Medicine, Catholic University St. Vincent Martyr of Valencia, Spain

§Mathematician with a master in Statistics, Department of Statistics, Research Foundation, Hospital General Universitario, Valencia, Spain

*Medical School, University College London, London, United Kingdom

CORRESPONDING AUTHOR

Professor V. Vanaclocha

University of Valencia

Avenida Blasco Ibañez 15, 46010 Valencia, SPAIN

Email: [vivava@uv.es](mailto:vivava@uv.es)
