# Supplementary material for: Selective T3–T4 sympathicotomy versus gray ramicotomy on outcome and quality of life in hyperhidrosis patients: a randomized clinical trial
Source: Sci Rep. 2021 Sep 2;11:17628. doi: 10.1038/s41598-021-96972-7 (PMC8413289; doi:10.1038/s41598-021-96972-7)
Supplement: Supplementary file 5 — Supplementary Information 5. [file 41598_2021_96972_MOESM5_ESM.docx]

| **Variable** | **Radicotomy** | **Sympathicotomy** | | **P-value** |  |
| --- | --- | --- | --- | --- | --- |
|  | 20 (50%) | 20 (50%) | |  |  |
| ***Forehead difference temp*** |  |  | | 0.130 |  |
| Mean (SD) | 0.17 (0.57) | 0.54 (0.88) | |  |  |
| Median (IR) | 0.10 (-0.15-0.40) | 0.25 (-0.03-0.92) | |  |  |
| ***Right-hand difference temp*** |  |  | | 0.420 |  |
| Mean (SD) | 0.32 (0.54) | 0.48 (0.69) | |  |  |
| Median (IR) | 0.30 (-0.03-0.60) | 0.35 (0.02-0.92) | |  |  |
| ***Left-hand difference temp*** |  |  | | 0.287 |  |
| Mean (SD) | 0.37 (0.56) | 0.58 (0.69) | |  |  |
| Median (IR) | 0.30 (0.00-0.53) | 0.60 (0.17-1.10) | |  |  |
| ***Left Axilla difference temp*** |  |  | | 0.091 |  |
| Mean (SD) | -0.02 (0.27) | 0.27 (0.69) | |  |  |
| Median (IR) | 0.00 (-0.20-0.13) | 0.00 (-0.10-0.10) | |  |  |
| ***Right Axilla difference temp*** |  |  | | 0.341 |  |
| Mean (SD) | 0.04 (0.30) | 0.24 (0.82) | |  |  |
| Median (IR) | 0.10 (-0.20-0.30) | 0.10 (-0.23-0.25) | |  |  |
| ***Abdomen difference temp*** |  |  | | **0.002** |  |
| Mean (SD) | 0.14 (0.35) | -0.27 (0.42) | |  |  |
| Median (IR) | 0.15 (-0.12-0.40) | -0.30 (-0.50–0.17) | |  |  |
| ***Right thigh difference temp*** |  |  | | **<0.001** |  |
| Mean (SD) | 0.60 (0.33) | 0.16 (0.34) | |  |  |
| Median (IR) | 0.60 (0.37-0.82) | 0.15 (-0.05-0.35) | |  |  |
| ***Left thigh difference temp*** |  |  | | **<0.001** |  |
| Mean (SD) | 0.49 (0.40) | -0.04 (0.49) | |  |  |
| Median (IR) | 0.50 (0.17-0.73) | -0.05 (-0.33-0.22) | |  |  |
| ***Right Foot difference temp*** |  |  | | **<0.001** |  |
| Mean (SD) | 0.46 (0.63) | -0.30 (0.44) | |  |  |
| Median (IR) | 0.45 (0.10-1.00) | -0.15 (-0.50-0.02) | |  |  |
| ***Left Foot difference temp*** |  |  | | **<0.001** |  |
| Mean (SD) | 0.38 (0.61) | -0.46 (0.77) | |  |  |
| Median (IR) | 0.45 (-0.05-0.75) | -0.50 (-0.93-0.18) | |  |  |
|  |  | |  | |  |
|  |  | |  | |  |
|  |  | |  | |  |

**Table S3**: Preoperative and postoperative July past one-year follow-up temperature data compared. A high value implies worsening, and a smaller value signifies an improvement. The temperature changes are milder for the gray rami communicantes group compared to the SY patients. These lesser temperature changes attest that the degree of sympathetic system lesion is gentler for the first than for the second group.

Selective T_3_-T_4_ sympathicotomy versus gray ramicotomy on outcome and quality of life in hyperhidrosis patients: a randomized clinical trial. Vicente Vanaclocha MD PhD&, Ricardo Guijarro-Jorge MD PhD♦, Nieves Saiz-Sapena MD PhD+, Manuel Granell-Gil MD PhD+, José María Ortiz-Criado MD PhD#, Juan Manuel Mascarós§, Leyre Vanaclocha BsC*

&Department of Neurosurgery, Hospital General Universitario de Valencia and Department of Surgery, Faculty of Medicine, University of Valencia, Valencia, Spain

♦Department of Thoracic Surgery, Hospital General Universitario de Valencia and Department of Surgery, Faculty of Medicine, University of Valencia, Valencia, Spain

+Department of Anesthesiology, Hospital General Universitario de Valencia, Valencia, Spain

#Instituto de Medicina Legal de Valencia (IMLV) and Department of Anatomy, Faculty of Medicine, Catholic University St. Vincent Martyr of Valencia, Spain

§Mathematician with a master in Statistics, Department of Statistics, Research Foundation, Hospital General Universitario, Valencia, Spain

*Medical School, University College London, London, United Kingdom

CORRESPONDING AUTHOR

Professor V. Vanaclocha

University of Valencia

Avenida Blasco Ibañez 15, 46010 Valencia, SPAIN

Email: [vivava@uv.es](mailto:vivava@uv.es)
