# Supplementary material for: Selective T3–T4 sympathicotomy versus gray ramicotomy on outcome and quality of life in hyperhidrosis patients: a randomized clinical trial
Source: Sci Rep. 2021 Sep 2;11:17628. doi: 10.1038/s41598-021-96972-7 (PMC8413289; doi:10.1038/s41598-021-96972-7)
Supplement: Supplementary file 6 — Supplementary Information 6. [file 41598_2021_96972_MOESM6_ESM.docx]

| **Variable** | **Ramicotomy** | **Sympathicotomy** | **P-value** |
| --- | --- | --- | --- |
|  | 20 (50%) | 20 (50%) |  |
| ***Forehead sweat baseline*** |  |  | 0.867 |
| Mean (SD) | 0.02 (0.01) | 0.02 (0.01) |  |
| Median (IR) | 0.02 (0.02-0.03) | 0.02 (0.02-0.03) |  |
| ***Right-hand sweat baseline*** |  |  | 0.146 |
| Mean (SD) | 0.16 (0.05) | 0.18 (0.06) |  |
| Median (IR) | 0.15 (0.13-0.18) | 0.18 (0.14-0.22) |  |
| ***Left-hand sweat baseline*** |  |  | 0.367 |
| Mean (SD) | 0.18 (0.06) | 0.20 (0.07) |  |
| Median (IR) | 0.17 (0.14-0.21) | 0.20 (0.14-0.25) |  |
| ***Left Axilla sweat baseline*** |  |  | 0.839 |
| Mean (SD) | 0.18 (0.07) | 0.17 (0.08) |  |
| Median (IR) | 0.16 (0.14-0.20) | 0.14 (0.12-0.19) |  |
| ***Right Axilla sweat baseline*** |  |  | 0.345 |
| Mean (SD) | 0.18 (0.05) | 0.20 (0.11) |  |
| Median (IR) | 0.16 (0.14-0.20) | 0.16 (0.13-0.22) |  |
| ***Abdomen sweat baseline*** |  |  | 0.863 |
| Mean (SD) | 0.03 (0.01) | 0.03 (0.02) |  |
| Median (IR) | 0.03 (0.02-0.04) | 0.03 (0.01-0.04) |  |
| ***Right thigh sweat baseline*** |  |  | 0.595 |
| Mean (SD) | 0.03 (0.01) | 0.03 (0.01) |  |
| Median (IR) | 0.03 (0.02-0.04) | 0.02 (0.02-0.03) |  |
| ***Left thigh sweat baseline*** |  |  | 0.707 |
| Mean (SD) | 0.03 (0.01) | 0.03 (0.01) |  |
| Median (IR) | 0.02 (0.02-0.03) | 0.03 (0.02-0.04) |  |
| ***Right Foot sweat baseline*** |  |  | 0.784 |
| Mean (SD) | 0.18 (0.06) | 0.18 (0.05) |  |
| Median (IR) | 0.16 (0.15-0.20) | 0.18 (0.14-0.20) |  |
| ***Left Foot sweat baseline*** |  |  | 0.574 |
| Mean (SD) | 0.17 (0.06) | 0.18 (0.04) |  |
| Median (IR) | 0.17 (0.14-0.19) | 0.18 (0.16-0.19) |  |

**Table S4:** Baseline quality of life and sweating according to the group. We measured the sweat in milligrams of water.

Selective T_3_-T_4_ sympathicotomy versus gray ramicotomy on outcome and quality of life in hyperhidrosis patients: a randomized clinical trial. Vicente Vanaclocha MD PhD&, Ricardo Guijarro-Jorge MD PhD♦, Nieves Saiz-Sapena MD PhD+, Manuel Granell-Gil MD PhD+, José María Ortiz-Criado MD PhD#, Juan Manuel Mascarós§, Leyre Vanaclocha BsC*

&Department of Neurosurgery, Hospital General Universitario de Valencia and Department of Surgery, Faculty of Medicine, University of Valencia, Valencia, Spain

♦Department of Thoracic Surgery, Hospital General Universitario de Valencia and Department of Surgery, Faculty of Medicine, University of Valencia, Valencia, Spain

+Department of Anesthesiology, Hospital General Universitario de Valencia, Valencia, Spain

#Instituto de Medicina Legal de Valencia (IMLV) and Department of Anatomy, Faculty of Medicine, Catholic University St. Vincent Martyr of Valencia, Spain

§Mathematician with a master in Statistics, Department of Statistics, Research Foundation, Hospital General Universitario, Valencia, Spain

*Medical School, University College London, London, United Kingdom

CORRESPONDING AUTHOR

Professor V. Vanaclocha

University of Valencia

Avenida Blasco Ibañez 15, 46010 Valencia, SPAIN

Email: [vivava@uv.es](mailto:vivava@uv.es)
