# Supplementary material for: Selective T3–T4 sympathicotomy versus gray ramicotomy on outcome and quality of life in hyperhidrosis patients: a randomized clinical trial
Source: Sci Rep. 2021 Sep 2;11:17628. doi: 10.1038/s41598-021-96972-7 (PMC8413289; doi:10.1038/s41598-021-96972-7)
Supplement: Supplementary file 7 — Supplementary Information 7. [file 41598_2021_96972_MOESM7_ESM.docx]

| **Variable** | **Radicotomy** | **Sympathicotomy** | **P-value** |
| --- | --- | --- | --- |
|  | 20 (50%) | 20 (50%) |  |
| ***Forehead temp baseline*** |  |  | 0.956 |
| Mean (SD) | 35.74 (0.46) | 35.73 (0.67) |  |
| Median (IR) | 35.85 (35.45-36.10) | 35.90 (35.58-36.10) |  |
| ***Right-hand temp baseline*** |  |  | 0.858 |
| Mean (SD) | 35.44 (0.49) | 35.41 (0.56) |  |
| Median (IR) | 35.45 (35.25-35.70) | 35.70 (35.10-35.80) |  |
| ***Left-hand temp baseline*** |  |  | 0.529 |
| Mean (SD) | 35.48 (0.42) | 35.39 (0.43) |  |
| Median (IR) | 35.50 (35.30-35.62) | 35.40 (35.20-35.52) |  |
| ***Left Axilla temp baseline*** |  |  | 0.976 |
| Mean (SD) | 36.05 (0.27) | 36.05 (0.68) |  |
| Median (IR) | 36.10 (35.88-36.10) | 36.25 (36.03-36.40) |  |
| ***Right Axilla temp baseline*** |  |  | 0.884 |
| Mean (SD) | 36.03 (0.24) | 36.05 (0.72) |  |
| Median (IR) | 36.05 (35.90-36.20) | 36.25 (35.98-36.50) |  |
| ***Abdomen temp baseline*** |  |  | 0.188 |
| Mean (SD) | 35.95 (0.26) | 36.09 (0.37) |  |
| Median (IR) | 35.95 (35.80-36.10) | 36.20 (36.00-36.30) |  |
| ***Right thigh temp baseline*** |  |  | 0.157 |
| Mean (SD) | 35.46 (0.27) | 35.60 (0.36) |  |
| Median (IR) | 35.50 (35.30-35.60) | 35.60 (35.30-35.90) |  |
| ***Left thigh temp baseline*** |  |  | 0.102 |
| Mean (SD) | 35.47 (0.40) | 35.68 (0.39) |  |
| Median (IR) | 35.50 (35.20-35.70) | 35.80 (35.20-36.00) |  |
| ***Right Foot temp baseline*** |  |  | 0.230 |
| Mean (SD) | 35.13 (0.62) | 35.34 (0.45) |  |
| Median (IR) | 34.90 (34.70-35.45) | 35.25 (35.10-35.55) |  |
| ***Left Foot temp baseline*** |  |  | 0.333 |
| Mean (SD) | 35.17 (0.71) | 35.38 (0.57) |  |
| Median (IR) | 34.90 (34.68-35.70) | 35.20 (34.88-35.82) |  |

**Table S5:** Baseline temperature per anatomical area according to the group measured in degrees Celsius.

| Selective T_3_-T_4_ sympathicotomy versus gray ramicotomy on outcome and quality of life in hyperhidrosis patients: a randomized clinical trial. Vicente Vanaclocha MD PhD&, Ricardo Guijarro-Jorge MD PhD♦, Nieves Saiz-Sapena MD PhD+, Manuel Granell-Gil MD PhD+, José María Ortiz-Criado MD PhD#, Juan Manuel Mascarós§, Leyre Vanaclocha BsC*  &Department of Neurosurgery, Hospital General Universitario de Valencia and Department of Surgery, Faculty of Medicine, University of Valencia, Valencia, Spain  ♦Department of Thoracic Surgery, Hospital General Universitario de Valencia and Department of Surgery, Faculty of Medicine, University of Valencia, Valencia, Spain  +Department of Anesthesiology, Hospital General Universitario de Valencia, Valencia, Spain  #Instituto de Medicina Legal de Valencia (IMLV) and Department of Anatomy, Faculty of Medicine, Catholic University St. Vincent Martyr of Valencia, Spain  §Mathematician with a master in Statistics, Department of Statistics, Research Foundation, Hospital General Universitario, Valencia, Spain  *Medical School, University College London, London, United Kingdom  CORRESPONDING AUTHOR  Professor V. Vanaclocha  University of Valencia  Avenida Blasco Ibañez 15, 46010 Valencia, SPAIN  Email: [vivava@uv.es](mailto:vivava@uv.es) |
| --- |
