# Supplementary material for: Selective T3–T4 sympathicotomy versus gray ramicotomy on outcome and quality of life in hyperhidrosis patients: a randomized clinical trial
Source: Sci Rep. 2021 Sep 2;11:17628. doi: 10.1038/s41598-021-96972-7 (PMC8413289; doi:10.1038/s41598-021-96972-7)
Supplement: Supplementary file 8 — Supplementary Information 8. [file 41598_2021_96972_MOESM8_ESM.docx]

| **Variable** | **Ramicotomy** | **Sympathicotomy** | **P-value** |
| --- | --- | --- | --- |
|  | 20 (50%) | 20 (50%) |  |
| ***Increase temp intraoperatively hand right*** |  |  | **0.012** |
| Mean (SD) | 0.59 (0.19) | 0.92 (0.51) |  |
| Median (IR) | 0.60 (0.48-0.70) | 0.90 (0.50-1.20) |  |
| ***Increase temp intraoperatively hand left*** |  |  | **0.016** |
| Mean (SD) | 0.62 (0.21) | 0.88 (0.41) |  |
| Median (IR) | 0.60 (0.48-0.80) | 0.85 (0.50-1.02) |  |
| ***Days admission*** |  |  | **0.042** |
| Mean (SD) | 1.15 (0.37) | 2.05 (1.82) |  |
| Median (IR) | 1.00 (1.00-1.00) | 1.00 (1.00-2.00) |  |

**Table S6:** Temperature measured intraoperatively in the thenar eminence rose more in the SY than in the gray rami communicantes lesion (RC). Postoperative stay length is longer for the SY group because they had one pneumothorax and another hemothorax.

Selective T_3_-T_4_ sympathicotomy versus gray ramicotomy on outcome and quality of life in hyperhidrosis patients: a randomized clinical trial. Vicente Vanaclocha MD PhD&, Ricardo Guijarro-Jorge MD PhD♦, Nieves Saiz-Sapena MD PhD+, Manuel Granell-Gil MD PhD+, José María Ortiz-Criado MD PhD#, Juan Manuel Mascarós§, Leyre Vanaclocha BsC*

&Department of Neurosurgery, Hospital General Universitario de Valencia and Department of Surgery, Faculty of Medicine, University of Valencia, Valencia, Spain

♦Department of Thoracic Surgery, Hospital General Universitario de Valencia and Department of Surgery, Faculty of Medicine, University of Valencia, Valencia, Spain

+Department of Anesthesiology, Hospital General Universitario de Valencia, Valencia, Spain

#Instituto de Medicina Legal de Valencia (IMLV) and Department of Anatomy, Faculty of Medicine, Catholic University St. Vincent Martyr of Valencia, Spain

§Mathematician with a master in Statistics, Department of Statistics, Research Foundation, Hospital General Universitario, Valencia, Spain

*Medical School, University College London, London, United Kingdom

CORRESPONDING AUTHOR

Professor V. Vanaclocha

University of Valencia

Avenida Blasco Ibañez 15, 46010 Valencia, SPAIN

Email: vivava@uv.es
