# Supplementary material for: Selective T3–T4 sympathicotomy versus gray ramicotomy on outcome and quality of life in hyperhidrosis patients: a randomized clinical trial
Source: Sci Rep. 2021 Sep 2;11:17628. doi: 10.1038/s41598-021-96972-7 (PMC8413289; doi:10.1038/s41598-021-96972-7)
Supplement: Supplementary file 9 — Supplementary Information 9. [file 41598_2021_96972_MOESM9_ESM.docx]

| \| **Variable** \| **Ramicotomy** \| **Sympathicotomy** \| **P-value** \| \| --- \| --- \| --- \| --- \| \|  \| 20 (50%) \| 20 (50%) \|  \| \| ***Forehead sweat POSTOP*** \|  \|  \| **0.003** \| \| Mean (SD) \| 0.04 (0.01) \| 0.02 (0.02) \|  \| \| Median (IR) \| 0.04 (0.03-0.04) \| 0.01 (0.01-0.02) \|  \| \| ***Right-hand sweat POSTOP*** \|  \|  \| **<0.001** \| \| Mean (SD) \| 0.06 (0.02) \| 0.03 (0.01) \|  \| \| Median (IR) \| 0.05 (0.04-0.07) \| 0.03 (0.02-0.04) \|  \| \| ***Left-hand sweat POSTOP*** \|  \|  \| **<0.001** \| \| Mean (SD) \| 0.06 (0.02) \| 0.03 (0.01) \|  \| \| Median (IR) \| 0.07 (0.05-0.08) \| 0.03 (0.02-0.04) \|  \| \| ***Left Axilla sweat POSTOP*** \|  \|  \| **<0.001** \| \| Mean (SD) \| 0.06 (0.01) \| 0.03 (0.02) \|  \| \| Median (IR) \| 0.06 (0.05-0.06) \| 0.03 (0.02-0.04) \|  \| \| ***Right Axilla sweat POSTOP*** \|  \|  \| **0.009** \| \| Mean (SD) \| 0.06 (0.01) \| 0.04 (0.03) \|  \| \| Median (IR) \| 0.06 (0.05-0.07) \| 0.03 (0.02-0.04) \|  \| \| ***Abdomen sweat POSTOP*** \|  \|  \| **0.006** \| \| Mean (SD) \| 0.02 (0.00) \| 0.12 (0.15) \|  \| \| Median (IR) \| 0.02 (0.02-0.02) \| 0.09 (0.08-0.12) \|  \| \| ***Right thigh sweat POSTOP*** \|  \|  \| **<0.001** \| \| Mean (SD) \| 0.02 (0.01) \| 0.09 (0.02) \|  \| \| Median (IR) \| 0.02 (0.02-0.03) \| 0.09 (0.08-0.10) \|  \| \| ***Left thigh sweat POSTOP*** \|  \|  \| **<0.001** \| \| Mean (SD) \| 0.02 (0.01) \| 0.09 (0.03) \|  \| \| Median (IR) \| 0.02 (0.01-0.03) \| 0.09 (0.08-0.09) \|  \| \| ***Right Foot sweat POSTOP*** \|  \|  \| **<0.001** \| \| Mean (SD) \| 0.05 (0.01) \| 0.14 (0.04) \|  \| \| Median (IR) \| 0.05 (0.04-0.05) \| 0.14 (0.12-0.15) \|  \| \| ***Left Foot sweat POSTOP*** \|  \|  \| **<0.001** \| \| Mean (SD) \| 0.05 (0.01) \| 0.15 (0.05) \|  \| \| Median (IR) \| 0.05 (0.04-0.05) \| 0.14 (0.12-0.16) \|  \| | | | | |  |  |  |  |
| --- | --- | --- | --- | --- | --- | --- | --- | --- | --- | --- | --- | --- | --- | --- | --- | --- | --- | --- | --- | --- | --- | --- | --- | --- | --- | --- | --- | --- | --- | --- | --- | --- | --- | --- | --- | --- | --- | --- | --- | --- | --- | --- | --- | --- | --- | --- | --- | --- | --- | --- | --- | --- | --- | --- | --- | --- | --- | --- | --- | --- | --- | --- | --- | --- | --- | --- | --- | --- | --- | --- | --- | --- | --- | --- | --- | --- | --- | --- | --- | --- | --- | --- | --- | --- | --- | --- | --- | --- | --- | --- | --- | --- | --- | --- | --- | --- | --- | --- | --- | --- | --- | --- | --- | --- | --- | --- | --- | --- | --- | --- | --- | --- | --- | --- | --- | --- | --- | --- | --- | --- | --- | --- | --- | --- | --- | --- | --- | --- | --- | --- | --- | --- | --- | --- | --- | --- |
|  |  |  |  |  |  |  |  |  |

**Table S7:** Postoperative sweating changes in different anatomical areas during July. Patients undergoing SY reported worse results than RC ones. Patients in the SY group sweated less in the hands, axillae, and forehead but much more than they used to in the abdomen, thighs, and feet. This extra sweat in areas where they did not sweat so much means that they suffer from worse CH.

Selective T_3_-T_4_ sympathicotomy versus gray ramicotomy on outcome and quality of life in hyperhidrosis patients: a randomized clinical trial. Vicente Vanaclocha MD PhD&, Ricardo Guijarro-Jorge MD PhD♦, Nieves Saiz-Sapena MD PhD+, Manuel Granell-Gil MD PhD+, José María Ortiz-Criado MD PhD#, Juan Manuel Mascarós§, Leyre Vanaclocha BsC*

&Department of Neurosurgery, Hospital General Universitario de Valencia and Department of Surgery, Faculty of Medicine, University of Valencia, Valencia, Spain

♦Department of Thoracic Surgery, Hospital General Universitario de Valencia and Department of Surgery, Faculty of Medicine, University of Valencia, Valencia, Spain

+Department of Anesthesiology, Hospital General Universitario de Valencia, Valencia, Spain

#Instituto de Medicina Legal de Valencia (IMLV) and Department of Anatomy, Faculty of Medicine, Catholic University St. Vincent Martyr of Valencia, Spain

§Mathematician with a master in Statistics, Department of Statistics, Research Foundation, Hospital General Universitario, Valencia, Spain

*Medical School, University College London, London, United Kingdom

CORRESPONDING AUTHOR

Professor V. Vanaclocha

University of Valencia

Avenida Blasco Ibañez 15, 46010 Valencia, SPAIN

Email: [vivava@uv.es](mailto:vivava@uv.es)
