# Supplementary material for: Selective T3–T4 sympathicotomy versus gray ramicotomy on outcome and quality of life in hyperhidrosis patients: a randomized clinical trial
Source: Sci Rep. 2021 Sep 2;11:17628. doi: 10.1038/s41598-021-96972-7 (PMC8413289; doi:10.1038/s41598-021-96972-7)
Supplement: Supplementary file 10 — Supplementary Information 10. [file 41598_2021_96972_MOESM10_ESM.docx]

| **Variable** | **Radicotomy** | **Sympathicotomy** | **P-value** | | |
| --- | --- | --- | --- | --- | --- |
|  | 20 (50%) | 20 (50%) | |  |  |
| ***Forehead temp POSTOP*** |  |  | | **<0.001** |  |
| Mean (SD) | 35.91 (0.26) | 36.27 (0.33) | |  |  |
| Median (IR) | 35.90 (35.70-36.20) | 36.20 (36.00-36.42) | |  |  |
| ***Right-hand temp POSTOP*** |  |  | | 0.195 |  |
| Mean (SD) | 35.76 (0.25) | 35.88 (0.36) | |  |  |
| Median (IR) | 35.80 (35.58-35.90) | 35.95 (35.60-36.10) | |  |  |
| ***Left-hand temp POSTOP*** |  |  | | 0.269 |  |
| Mean (SD) | 35.84 (0.33) | 35.97 (0.40) | |  |  |
| Median (IR) | 35.80 (35.68-35.92) | 36.00 (35.77-36.30) | |  |  |
| ***Left Axilla temp POSTOP*** |  |  | | **<0.001** |  |
| Mean (SD) | 36.02 (0.15) | 36.31 (0.18) | |  |  |
| Median (IR) | 36.00 (35.90-36.10) | 36.30 (36.20-36.40) | |  |  |
| ***Right Axilla temp POSTOP*** |  |  | | **0.004** |  |
| Mean (SD) | 36.08 (0.21) | 36.29 (0.23) | |  |  |
| Median (IR) | 36.10 (35.90-36.30) | 36.30 (36.10-36.50) | |  |  |
| ***Abdomen temp POSTOP*** |  |  | | **<0.001** |  |
| Mean (SD) | 36.09 (0.24) | 35.81 (0.14) | |  |  |
| Median (IR) | 36.15 (35.90-36.30) | 35.85 (35.70-35.90) | |  |  |
| ***Right thigh temp POSTOP*** |  |  | | **<0.001** |  |
| Mean (SD) | 36.06 (0.17) | 35.76 (0.18) | |  |  |
| Median (IR) | 36.10 (35.90-36.20) | 35.80 (35.68-35.90) | |  |  |
| ***Left thigh temp POSTOP*** |  |  | | **<0.001** |  |
| Mean (SD) | 35.96 (0.22) | 35.64 (0.27) | |  |  |
| Median (IR) | 35.95 (35.80-36.10) | 35.70 (35.38-35.80) | |  |  |
| ***Right Foot temp POSTOP*** |  |  | | **<0.001** |  |
| Mean (SD) | 35.59 (0.27) | 35.05 (0.44) | |  |  |
| Median (IR) | 35.60 (35.38-35.82) | 35.20 (34.98-35.30) | |  |  |
| ***Left Foot temp POSTOP*** |  |  | | **<0.001** |  |
| Mean (SD) | 35.55 (0.28) | 34.91 (0.55) | |  |  |
| Median (IR) | 35.60 (35.30-35.73) | 35.10 (34.58-35.30) | |  |  |

**Table S8**: Postoperative temperature changes in different anatomical areas. SY patients had a more significant rise in the forehead, with a colder temperature in the abdomen, thighs, and soles of feet than in the RY gray rami communicantes group. These temperature changes indicate that SY induces a more prominent sympathetic system lesion and that the gray rami communicantes RY causes a more selective lesion with fewer side effects.

| Selective T_3_-T_4_ sympathicotomy versus gray ramicotomy on outcome and quality of life in hyperhidrosis patients: a randomized clinical trial. Vicente Vanaclocha MD PhD&, Ricardo Guijarro-Jorge MD PhD♦, Nieves Saiz-Sapena MD PhD+, Manuel Granell-Gil MD PhD+, José María Ortiz-Criado MD PhD#, Juan Manuel Mascarós§, Leyre Vanaclocha BsC*  &Department of Neurosurgery, Hospital General Universitario de Valencia and Department of Surgery, Faculty of Medicine, University of Valencia, Valencia, Spain  ♦Department of Thoracic Surgery, Hospital General Universitario de Valencia and Department of Surgery, Faculty of Medicine, University of Valencia, Valencia, Spain  +Department of Anesthesiology, Hospital General Universitario de Valencia, Valencia, Spain  #Instituto de Medicina Legal de Valencia (IMLV) and Department of Anatomy, Faculty of Medicine, Catholic University St. Vincent Martyr of Valencia, Spain  §Mathematician with a master in Statistics, Department of Statistics, Research Foundation, Hospital General Universitario, Valencia, Spain  *Medical School, University College London, London, United Kingdom  CORRESPONDING AUTHOR  Professor V. Vanaclocha  University of Valencia  Avenida Blasco Ibañez 15, 46010 Valencia, SPAIN  Email: [vvanaclo@hotmail.com](mailto:vvanaclo@hotmail.com) |
| --- |
